# The Anti-Epileptic Effects of Carbenoxolone In Vitro and In Vivo

**DOI:** 10.3390/ijms23020663

**Published:** 2022-01-08

**Authors:** Anna Volnova, Vassiliy Tsytsarev, Olga Ganina, Grace E. Vélez-Crespo, Janaina M. Alves, Alla Ignashchenkova, Mikhail Inyushin

**Affiliations:** 1 Biological Faculty, Saint Petersburg State University, 199034 St. Petersburg, Russia; 2 Institute of Translational Biomedicine, Saint Petersburg State University, 199034 St. Petersburg, Russia; alla.ignashchenkova@gmail.com; 3 School of Medicine, University of Maryland, Baltimore, MD 21201, USA; tsytsarev@umaryland.edu; 4 Nevsky Center of Scientific Collaboration, 192119 St. Petersburg, Russia; olga.ganina92@mail.ru; 5 School of Medicine, Universidad Central del Caribe, Bayamon, PR 00956, USA; 416gvelez@uccaribe.edu (G.E.V.-C.); janaina.alves@uccaribe.edu (J.M.A.)

**Keywords:** astrocytes, carbenoxolone, epilepsy, gap junction, neural synchronization, seizures

## Abstract

Gap junctions (GJs) are intercellular junctions that allow the direct transfer of ions and small molecules between neighboring cells, and GJs between astrocytes play an important role in the development of various pathologies of the brain, including regulation of the pathological neuronal synchronization underlying epileptic seizures. Recently, we found that a pathological change is observed in astrocytes during the ictal and interictal phases of 4-aminopyridin (4-AP)-elicited epileptic activity in vitro, which was correlated with neuronal synchronization and extracellular epileptic electrical activity. This finding raises the question: Does this signal depend on GJs between astrocytes? In this study we investigated the effect of the GJ blocker, carbenoxolone (CBX), on epileptic activity in vitro and in vivo. Based on the results obtained, we came to the conclusion that the astrocytic syncytium formed by GJ-associated astrocytes, which is responsible for the regulation of potassium, affects the formation of epileptic activity in astrocytes in vitro and epileptic seizure onset. This effect is probably an important, but not the only, mechanism by which CBX suppresses epileptic activity. It is likely that the mechanisms of selective inhibition of GJs between astrocytes will show important translational benefits in anti-epileptic therapies.

## 1. Introduction

Epilepsy is a widespread disease of the central nervous system resulting from abnormal neural synchronization, which causes recurring seizures spreading across the brain. Its main feature is the sudden onset of synchronized activity of many neurons, leading to various pathological psychomotor manifestations [1]. Today, pharmacological agents, diet, deep brain stimulation, and, in severe cases, neurosurgical treatment are used for antiepileptic therapy. Despite great progress in the development of new anti-epileptic drugs, a significant number of cases of epilepsy are pharmacoresistant. In addition, many antiepileptic drugs have significant side effects. Thus, there is an urgent need for further study of epileptogenesis and the development of new antiepileptic drugs.

It is well known that GJs play a key role in the normal and pathological synchronization of neuronal circuits and the astrocyte syncytium. They consist of half channels (connexons), formed by adjacent cells from each side of the contact, and when united, form a channel (connexin) between cells. Each connexon, in turn, consists of six protein subunits known as connexins [2]. From a physiological point of view, GJs are intercellular contacts between cells that allow ions and small, biologically active molecules to pass directly from one cell to another. Such movement is often associated with the transfer of electric charge, which carries out electrical conduction. GJ permeability is variable and is regulated by cellular pH, calcium ion concentration, and a number of other biochemical factors [2,3]. It can also be controlled by special chemical agents with different mechanisms of action. One of the important conditions affecting GJ opening is pH, and there is clear acid-induced uncoupling of GJs [4]. Such uncoupling is important, because during neuronal synchronization there can be profound pH shifts [5]. Genetically inserted optical pH sensors in mouse brain have shown that astrocytes react to epileptiform activity with intracellular alkalization and augmented coupling [6], and similar augmented coupling was reported for astrocyte GJs during epilepsy [7]. Many studies in recent years have shown that brain GJs are involved in the generation, synchronization, and maintenance of epileptic activity [8,9], and there is evidence that GJ blockers have potent therapeutic potential in epilepsy.

The hypothesis that GJs play a significant role in the underlying mechanisms of epileptic seizures is quite popular. Among other arguments in favor of this hypothesis, epileptic activity is usually determined by excessive neural synchronicity, and GJs increase synchronicity. Recently, evidence has accumulated that the GJ inhibitor CBX regulates neuronal synchronization and suppresses epileptiform activity in the brain [10,11,12,13].

Recently, we described periodic signals in the astrocyte syncytium during the interictal phase of 4-AP-elicited epileptic seizures, and these signals were correlated with synchronized neuronal spikes, while ictal events were correlated with profound depolarization of the astrocyte syncytium and with vasomotion [14]. It remains unclear whether these signals depend on GJs between astrocytes and how blocking these channels correlates with the synchronization of neurons.

The easiest way to block GJs is with medication, and the most commonly used blocker is CBX, which is modestly effective and water soluble [12]. CBX reduces seizure-related synchronization in various model systems, both in vivo and in vitro. However, CBX blocks both astrocytic GJs and neuronal GJs. The anticonvulsant effects of CBX and other GJ blockers, thus, are often interpreted as evidence that electrical contacts between neurons play a critical role in the onset of seizures, and direct measurements in astrocytes would therefore be of special interest. In this study we investigated the effect of the GJ blocker CBX on epileptic activity in astrocytes in vitro, induced with the widely used potassium channel inhibitor 4-AP, and the corresponding epileptic electrical activity in vivo [2].

## 2. Results

### 2.1. In Vitro Results

#### 2.1.1. Depolarizing Inward Currents Synchronized with the Epileptic Activity of Neurons Is Observed in Astrocytes

**Figure 1:**
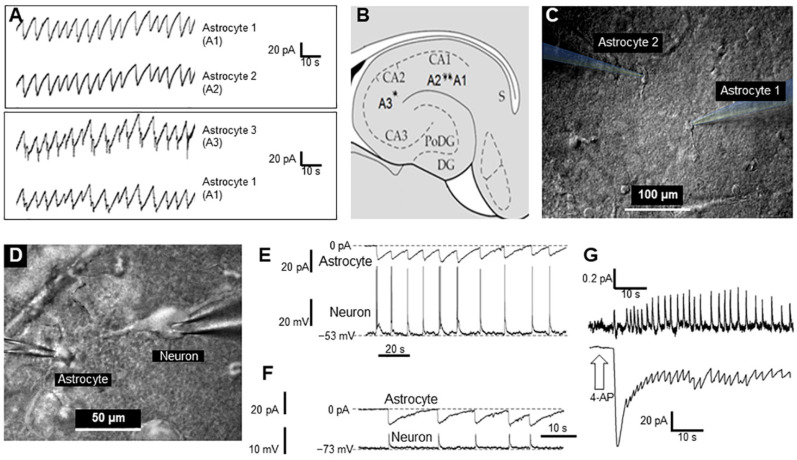
Synchronous activity of two astrocytes and neurons in the CA1 zone of the rat hippocampus after short-term application of 2 mM 4-AP: (**A**). Currents recorded from hippocampal astrocytes 1 and 2 (top panel) and 1 and 3 (bottom panel). (**B**). Sketch of the hippocampus with schematic positions of the astrocytes that were monitored in pairs. (**C**). Infrared image of two astrocytes with electrodes for patch-clamp. (**D**). Infrared image of a neuron and an astrocyte with electrodes attached for patch-clamp. (**E**,**F**). Current and voltage recording from an astrocyte and neuron, respectively. Panel E shows the neuron action potentials (APs), and the synchronous changes in the astrocyte’s membrane potential (Panel F) shows the excitatory postsynaptic potentials (EPSPs) of the neuron, observed during artificial hyperpolarization of the neuron, and synchronous changes in the membrane potential of an astrocyte. (**G**). Extracellular recording of neuron firing and current changes recorded from an astrocyte (top and bottom, respectively) elicited by 4-AP.

Using a dual-channel patch clamp in voltage clamp mode, we recorded membrane current in two quiescent astrocytes simultaneously. As we described previously, after perfusion of the slice preparation for 30–60 s with 2 mM 4-AP dissolved in ACSF, the inward current oscillations started to appear in astrocytes, with a mean amplitude of 37.7 ± 2.3 pA in a single astrocyte at 0.1–0.3 Hz (*n* = 27). Besides these small inward currents, short (2–3 s) but high-voltage (40–50 mV) depolarization events perturbed astrocytes periodically [14]. Here we present a similar series of experiments (*n* = 10) that were performed on patch pairs of astrocytes at different distances (20–600 µM) from each another. In the latter case, one astrocyte was situated in the CA1 zone and the second in the CA2 zone of the hippocampus (Figure 1A–C). Remarkably, there was near synchronization of periodically generated inward currents in nearby astrocytes as well as in distant astrocytes, which suggests a relatively large spatial extent (≥0.6 mm) of the synchronization phenomenon. To illustrate this synchronization, the currents recorded from two pairs of astrocytes are presented in Figure 1.

We also monitored neuronal activity during 4-AP application, and this was not blocked. Figure 1D,E shows a typical simultaneous recording of an astrocyte (voltage-clamp mode) and neuron (current-clamp mode), each with glass electrodes. Interestingly, activity spikes in neurons corresponded to the inward currents in astrocytes (Figure 1E). 

Even when inhibiting neuronal spiking by applying a hyperpolarization current, giant excitatory synaptic currents were recorded in neurons, which were synchronized with inward current activity in astrocytes (Figure 1F). This suggests that astrocytes (and their syncytiums) respond with visible inward currents to potassium released by neurons during synchronized spikes. Moreover, we see that a majority of neurons (86% of all tested, *n* = 120) are driven by high excitatory synaptic potentials. Other neurons (14%) had no excitatory drive after application of 4-AP (not shown). 

Interestingly, inward currents in astrocytes (voltage-clamp mode, Figure 1G, lower trace) corresponded to the extracellular local field potentials (LFPs) in the slice (Figure 1G, upper trace). This finding suggests that global synchronization of both neurons and astrocytes produces LFP signals in the slice and probably in whole brain. 

#### 2.1.2. CBX Inhibits GJs in Astrocytes and Blocks Epileptiformic Activity in Astrocytes in Hippocampal Slices In Vitro

**Figure 2:**
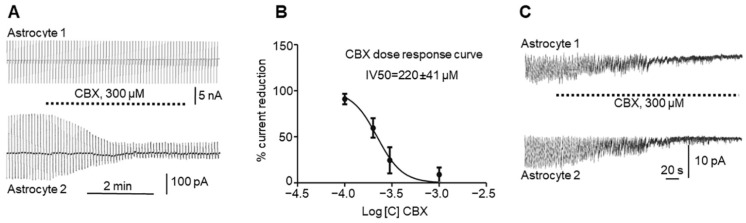
Effects of CBX on astrocyte gap junctions and on astrocyte activity elicited by 4-AP: (**A**). Reduction in gap junction current between astrocytes (lower curve) by application of 300 µM CBX to the bath (see text). (**B**). Dose–response curve of the CBX effect on inter-astrocyte gap junctions, with error bars representing SD. (**C**). CBX at a 300 µM concentration simultaneously reduces astrocytic “epileptiform” activity elicited by 4-AP in two astrocytes in the hippocampal slice.

We recorded GJ currents in astrocyte pairs connected by GJs (*n* = 16, Figure 2A) in control animals (see Methods). We injected current in the first astrocyte of the pair (astrocyte 1, upper trace) and recorded the current that passed through the GJs to the second astrocyte in the pair (astrocyte 2, lower trace). After perfusion of the slice with 300 µM CBX (Figure 2A), the effect was a significant reduction in current in the adjacent astrocyte, which was reduced after CBX perfusion (from 284.3 ± 58.9 to 34.2 ± 7.1; paired *t* test, *p* = 0.0058) but not completely blocked. Usually, the gap current was not restored after CBX application and washout and remained low. 

We also used different concentrations of CBX and paired recordings in astrocytes to build a dose–response curve (Figure 2B), showing that 220 ± 41 µM (*n* = 60) CBX applied to the slice reduced the number of functional GJs by half.

Interestingly, the astrocyte “epileptiform” activity that appeared during epileptic events was affected by CBX. After perfusion of the slice with 2 mM 4-AP for 30–60 s, an astrocyte inward current was initiated. The perfusion of the slice with 300 µM CBX blocked astrocyte epileptiform activity simultaneously in all recorded astrocytes (Figure 2C), which could be restored after washout by a new perfusion with 4-AP (again, this blocking and restoring of activity can be carried out more than once).

### 2.2. In Vivo Results

**Figure 3:**
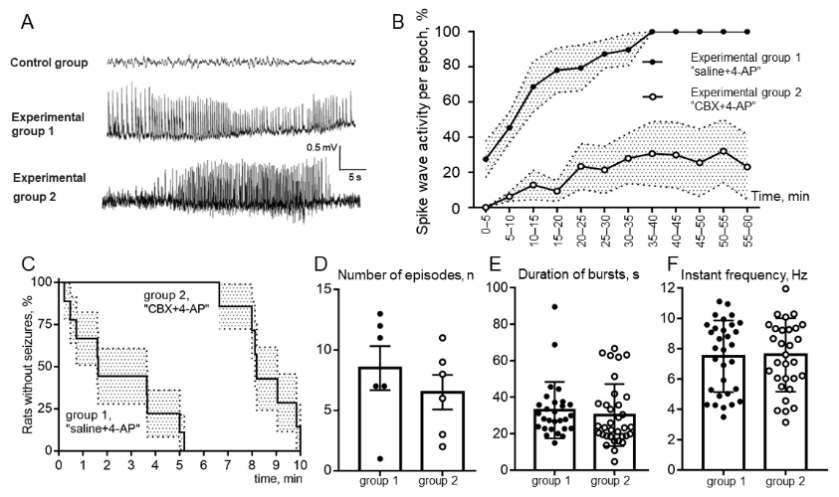
(**A**). Examples of a ECoG records; control group, no spike-wave activity (SWA) after only CBX or saline intracortical injection; group 1, ECoG of status epilepticus in a rat that received 4-AP after saline injection; group 2, seizure of epileptiform spike-wave activity on an ECoG of a rat that received 4-AP after CBX injection. (**B**). Comparison of the total duration of episodes of SWA over the epoch of analysis; (**C**). Percentage of animals of experimental groups 1 and 2 without seizure free during 10 min above 4-AP injection (latency to first SWA episode); (**D**). Comparison of the number of SWA episodes; (**E**). The mean values of the duration of bursts; (**F**). Comparison of instantaneous repetition rate in the composition of SWA episodes in rats receiving 4-AP after saline (group 1, “saline + 4-AP”) and in rats receiving 4-AP after administration of CBX (group 2, “CBX + 4-AP”). The abscissa shows the time in minutes. The mean values and errors of the mean are given, and the reliability of the differences was determined by two-way ANOVA, *p* < 0.0001 (**B**); The log-rank (Mantel–Cox) test, *p* < 0.0001 (**C**); the means ± SD, unpaired Mann–Whitney test, *p* > 0.05 (**D**–**F**).

Analysis of ECoG (Figure 3A) recordings showed that none of the animals receiving only saline or CBX (the control group) showed signs of epileptiform spike-wave activity (SWA). Analysis of the behavior of animals in the experimental box after the injection of these substances into the cerebral cortex also did not reveal any pathological manifestations of epileptic convulsive motor activity. In experiments after introduction of the epileptogen 4-AP, the development of epileptiform SWA was observed, but the degree of its manifestation was different in the two experimental groups. 

The rats of group 1, which had received intracortical microinjection of an isotonic NaCl solution before the induction of epilepsy, demonstrated more severe forms of epileptiform activity. Among the rats that received 4-AP after saline injection, 100% of the animals showed SWA, and 83% (5 of 6 animals) showed the development of status epilepticus. The rats of group 2, which had previously been injected with CBX, exhibited significantly fewer manifestations of experimental epilepsy. Thus, preliminary intracortical administration of CBX significantly reduced the severity of epileptiform activity after administration of 4-AP, with only 17% (1 rat of 6) demonstrating the development of status epilepticus (Figure 3A).

Comparison of the latent periods of the onset of episodes of SWA showed (Figure 3C) a significant increase in the latency of the first episode in rats receiving 4-AP after CBX compared with rats receiving 4-AP after saline. The significance of the differences was determined using the Mann–Whitney test: the average latency was 2.46 ± 0.65 min in group 1 and 8.55 ± 0.44 min in group 2. The analysis of the percentage of rats in experimental groups 1 and 2 remaining seizure-free demonstrated that rats after intracortical injection of “saline + 4-AP” have significantly shorter latent periods of episodes of SWA than rats in the “CBX + 4-AP” group (log-rank Mantel–Cox test, hazard ratio group 1/group 2–4.62; 95% CI of ratio –1.41 to 15.13; *p* < 0.0001).

The mean duration and the number of epileptic episodes was calculated without considering status epilepticus in animals of experimental group 1. The durations of SWA episodes (outside of status epilepticus) in rats in both experimental groups (Figure 3E) did not differ significantly (32.9 ± 2.9 s and 30.2 ± 2.9 s, respectively). The number of epileptic episodes also did not show a significant difference (Figure 3D). It should be noted that these parameters are not a quite representative parameter for comparing groups 1 and 2, because in rats of experimental group 1, some episodes of epileptic activity were transferred to status epilepticus, representing one single prolonged episode. At the same time, the number of episodes in rats of group 2 was relatively lower due to the increased latent period of the appearance of the first episode.

To estimate the percentage of time occupied by seizures, the ECoG recording after administration of 4-AP was divided into 5 min analysis periods. It was found that rats that received the 4-AP injection followed by saline reached 100% of the total duration of CBA episodes for the epoch of analysis 35 min, on average, after administration of 4-AP, which indicates the presence of status epilepticus (Figure 3B). The rats that received intracortical administration of CBX before induction of epilepsy did not reach even 40% of the level of the total duration of SWA episodes over the epoch of analysis. Comparison by two-way ANOVA showed that in rats receiving 4-AP after CBX, the total duration of SWA episodes was significantly lower than in rats receiving 4-AP after saline (Figure 3B), and these differences were significant at *p* < 0.0001.

A comparative analysis of the instantaneous repetition rate of spike waves in the composition of SWA episodes was carried out in rats of experimental groups 1 and 2 (Figure 3F). Statistical analysis using the nonparametric Mann–Whitney test showed that the instantaneous repetition rate of spike waves in the composition of SWA episodes did not differ between the analyzed groups of animals (7.51 ± 0.42 versus 7.59 ± 0.43). Comparison by the method of two-way ANOVA showed that the repetition rate of spike waves in the composition of seizures was not dependent on the epoch of analysis. Thus, it was shown that intracortical microinjections of CBX 30 min before the formation of an epileptic focus did not affect the instantaneous repetition rate of spike waves.

## 3. Discussion

Neurons and astrocytes interact during normal and pathological brain functioning. Besides providing trophic and structural support, astrocytes play an essential role in normal synaptic function, including synaptogenesis, synaptic transmission, and the modulation of neuronal network activity, mainly through their uptake of GABA and glutamate. Moreover, the astrocyte syncytium functions as a potassium (K^+^) spatial clamp, which also modulates neuronal excitability [15,16,17,18]. Many scientists believe that electrical coupling mediated by GJs plays an important role in brain pathologies, including abnormal synchronization, which underlies epileptic seizures [10,11,12,13]. 

4-AP-induced synchronous activity in epileptic foci has been observed not only in neurons but also in astrocytes [14]. Most studies associate this activity with the massive release of potassium from neurons into the extracellular space. The membrane potential of astrocytes changes proportionately less than the membrane potential of neurons, due to the stabilizing effect of the astrocyte syncytium. However, during epileptic activity, neuronal spikes become synchronous, and the neurons release K^+^ synchronously, thus overcoming this stabilizing (clamping) effect. The astrocytes then start to show depolarization activity, producing even more synchronization [14]. 

According to our hypothesis, CBX does not suppress the epileptic activity of neurons directly, but it blocks the GJs between astrocytes, which suppresses their synchronization. We observed in our in vitro experiments that stimulation of one astrocyte leads to membrane potential changes in an associated astrocyte through GJs. 

In this study we investigated in vitro whether GJs between astrocytes participate in epileptic seizures elicited by 4-AP and whether the GJ blocker CBX reduces both GJ electrical conductivity and seizures. In rat hippocampal slices, 4-AP produces periodic sawtooth inward currents that are nearly synchronous in all astrocytes in the slice (Figure 1A), as well as with the epileptic activity of neurons (Figure 1E,F) and slice extracellular local field potentials (LFPs, Figure 1G). CBX blocks this epileptiformic activity in astrocytes in hippocampal slices (Figure 2C) and reduces GJ conductivity in astrocytes (Figure 2A). Interestingly, neuronal synchronization was also eliminated by CBX.

The main object of our study was the interaction of astrocytes and neurons based on gap junctions during epileptogenesis [15]. Astrocytes and gap junctions are widely represented in both the cortex and the hippocampus, but living hippocampal slices have significant advantages [16]. A major advantage of hippocampal slice preparations is that the cytoarchitecture and synaptic circuits of the hippocampus are largely retained. The hippocampal brain slice proved to be particularly suitable because much of the intrinsic circuitry remains intact in a transverse slice. Because of its lamellar organization, much of the trisynaptic circuit remains intact in a transverse hippocampal slice [18]. Hippocampal living slices are most convenient for many neurophysiological studies, including our study, which require recording of neural and astrocytic activity. On the other hand, the 4-AP model of focal epileptic seizures in the cerebral cortex is well established, reliable, and allows the study of neuronal–astrocytic interactions in vivo [14,17]. The cerebral cortex contains a large number of astrocytes and allows easy local administration of any agents including 4-AP and CBX from the dorsal surface. Such administration is not required histological control. Thus, intracortical administration of 4-AP was the most appropriate for in vivo experiments. In in vitro experiments the use of surviving hippocampal slices was the most appropriate. 

During in vivo experiments we administered 4-AP intracortically, waited for extracellularly recorded epileptiform spike-wave activity (SWA) episodes, and injected CBX into the same epileptic zone. Experiments showed that the latent periods of the onset of episodes of SWA in rats were significantly increased by CBX (Figure 3C), while the total duration of episodes of SWA over the epoch of analysis were greatly reduced by CBX (Figure 3B). The other parameters of epileptiform activity (mean durations and the number of episodes of SWA, Figure 3D,E) were not quite representative because in rats of experimental group 1 some episodes of epileptic activity were transferred to status epilepticus (Figure 3A). At the same time, it was shown that the instantaneous repetition rate of SWA (Figure 3F) did not depend on intracortical microinjections of saline or CBX and has been completely determined by microinjection of 4-AP. These in vitro and in vivo results, taken together, suggest the possible involvement of astrocytic GJs in “in vivo” epileptiform activity in astrocytes and (indirectly, through space clamp) in neurons.

Epileptic activity leads to an increase in extracellular potassium concentration. Using the 4-AP-induced acute seizure model, it has been repeatedly investigated how extracellular potassium modulates epileptic seizure both in vivo and in vitro. It has been observed that moderately elevated potassium increases the duration of seizures and shortens interictal intervals, as well as depolarizing the resting membrane potential of neurons. However, when the extracellular potassium concentration reaches a certain value, paroxysmal events are blocked, and neurons become depolarized. This concentration-dependent dual effect is observed in vivo and in vitro, as well as in human neocortical tissue resected during epilepsy surgery [19]. 

**Figure 4:**
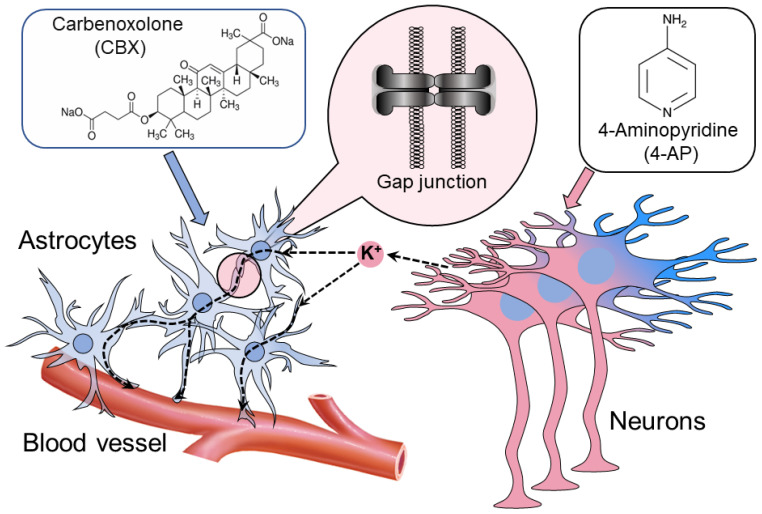
Movement of K^+^ (dotted arrows) in the astrocytic syncytium and their interaction with neural circuits. Gap junctions play a crucial role in the dispersal of K^+^ and its buffering by astrocytes. The astrocytic syncytium evens out the concentration of extracellular K^+^, absorbing it in areas of increased concentration and secreting it in areas of low concentration. Individual astrocytes reduce the concentration of K^+^ by absorbing it through the Kir1.4 channel, and excess K^+^ is released into the bloodstream. Intracortically injected 4-AP acts on neurons, causing synchronized epileptiform activity.

Summarizing in one scheme (Figure 4), astrocytes interconnected by GJs disperse the excessive release of neuronal K^+^ (the uptake is concentrated in the excessive K^+^ zone and the release in the less-depolarized zone), thus balancing extracellular K^+^ and releasing the excess through astrocyte endfeet enwrapping the vessel [18,20]. During epileptic synchronization, this clamping activity of the astrocytes becomes impossible because of the synchronous release of K^+^ at all sites simultaneously. This leads to the uncontrollable accumulation of K^+^ extracellularly and local depolarization of neurons, producing synchronous ictal seizure. To reignite the K^+^ space clamp, the astrocytes must be disconnected with GJ blockers.

It is possible that these effects of CBX depend not only on its ability to inhibit GJs but also on some other, as yet unknown, properties. GJ blockers, including CBX, are not highly selective. Thus, CBX induces reversible suppression of spontaneous discharges of action potentials, synaptic currents, and synchronized calcium oscillations in hippocampal neurons [21]. CBX also inhibits oscillatory activity induced by the GABA antagonist bicuculline. All of these effects have been shown to be unrelated to astrocyte GJs. 

It can be assumed that CBX acts not only on astrocytic but also on interneuronal GJs [22,23]. This conclusion was made on the basis of studies in which quinine or mefloquine were used in parallel with CBX, which are more selective and specific blockers of neuronal connexins, in particular of Cx36 [12,22,24]. All of these blockers have similar effects both in vivo and in vitro. Since Cx36 is expressed predominantly in GABAergic interneurons, it is possible that GJ inhibitors disrupt the inhibitory activity of neurons, and thereby suppress epileptic activity [23,25]. 

Unfortunately, GJs between neurons can be affected by CBX as well, presenting a chicken-and-egg problem and offering no explanation as to the initial cause of epileptic synchronicity in neurons or astrocytes. Moreover, CBX can affect chemical synaptic transmission as well. Thus, it was shown that in coronary thalamocortical slices of Wistar rats, paired impulse depression (PPD) is reduced by CBX [18]. This probably speaks to the effect of CBX directly on synaptic transmission, and this effect could contribute to the antiepileptic properties of CBX [18]. 

However, it should be remembered that there are no pharmacological agents that strictly affect only one link in numerous intercellular interactions, and CBX is no exception. The physiological activity of CBX is probably not limited to GJ inhibition, and we cannot yet rule out the possibility that CBX also affects synaptic transmission. At the same time, it is known that the elimination of specific interneurons probably causes disruption of inhibitory processes in the cortex [13]. The opposite can also be assumed: a specific intervention in certain parts of neural circuits can effectively suppress epileptic activity. The concentration of extracellular potassium indiscriminately affects synaptic transmission, but the sensitivity of different neurons to extracellular potassium is different. Among other things, this is most likely a consequence of the difference in the spread of epileptic activity at different potassium concentrations [26,27]. GABAergic interneurons seem to play a key role in these processes. However, they manifest themselves differently in different neural circuits [1,28,29]. GABAergic neurons can have both inhibitory and shunting properties, and the manifestation of these properties depends on many factors [1,30]. The possible impact of CBX on these processes is unclear and requires more research.

Another key role of the interaction between neurons and astrocytes in the formation of an epileptic focus was shown in a comprehensive study on guinea pigs [28,29]. It has been demonstrated that a group of neurons-carriers of pathological activity, activates astrocytes, which leads to the formation of an ictal event. At the same time, astrocytes likely play a key role in initiating seizures, not only in pathological, but also in normal brain tissue. This specificity of neuroglial interaction makes it the main target for the development of new pharmacological agents aimed at selectively interfering with ictogenesis.

On the cellular level it was also shown that the proteins of the gap junction subunits connexin 43 and 30 provide the transport of glucose and its metabolites through the astrocytic syncytium. This glucose transport is regulated by AMPA receptor-mediated glutamatergic synaptic activity. A low level of extracellular glucose causes activation of the delivery of glucose or lactate to astrocytes, which, in the presence of working gap junction, supports glutamatergic synaptic transmission and epileptiform activity [31].

There is a well-founded hypothesis that gap junctions of astrocytes contribute to the spatial buffering of potassium [31]. Genetically modified mice deficient in connexin 43 and connexin 30 are a convenient model to explore this concept [31]. It has been demonstrated that gap junctions between astrocytes accelerate potassium clearance and limit potassium accumulation during synchronized excitation of neurons [32]. In the surviving brain slices of such mice, a reduced threshold for the generation of epileptiform events is observed. In addition, an abnormally high astrocyte capacity for K+ is retained in mice with gap junctions deficient between astrocytes, indicating that gap junction-dependent processes only partially explain potassium buffering [31,32].

Genetically modified mice with inactivation of the Tsc1 gene in astrocytes show reduced expression connexin, Cx43. This leads to the pathology of gap junctions between astrocytes. Experiencing sections of the hippocampus of these mice showed an increased concentration of extracellular potassium in response to stimulation [33]. This impairment of potassium buffering is likely due to abnormal astrocytic gap junctions, since the gap junction inhibitor causes an additional increase in potassium concentration in controls, but not in genetically modified animals. In this way, inactivation of the Tsc1 gene in astrocytes causes defects in astrocytic gap junctions and potassium clearance, which may contribute to the development of epilepsy in this strain of mice [33].

Evidence was received that the disruption of intercellular communication through gap junctions of astrocytes is a key event in epileptogenesis. Hyperthermia (HT) causes seizures in mice, as well as a decrease in the expression of the protein connexin 43 and, accordingly, a decrease in gap junctions between astrocytes in the hippocampus by more than 50%. This process is not accompanied by the death of neurons and astrocytes [34].

Finally, it was shown that, possibly, in a 4-AP model of epilepsy in vitro, disruption of the connexin 36 is not critical for the generation of epileptiform discharges in GABA-ergic neural circuit. Hence, the antiepileptic effects of CBX may be directly due to blockade of GABA receptors rather than connexin 36-based gap junction inhibition [34].

Many forms of epilepsy are associated with disruption of GABAergic interneurons responsible for inhibition within the cortical neural circuits. Many GABAergic neurons are interconnected by gap junctions that can be effectively blocked by CBX. If this is the case, then, in all likelihood, CBX reduces the activity of inhibitory neurons and thereby intensifies the generation of synchronized epileptiform discharges. Thus, perhaps the effect of CBX is dual in nature: it supports epileptiform activity by inactivating GABAergic inhibitory neurons, and, at the same time, induces an antiepileptic effect through other cellular mechanisms. Moreover, there was evidence that the presence of connexin 36 is not a critical requirement for 4-AP-induced epileptiform interneuron synchronization [35]. It has been demonstrated that the antiepileptic effect of CBX on 4-AP-induced epileptiform activity may be caused, not by connexin 36-based gap junction blockade, but by direct action on postsynaptic GABA-A receptors [35]. These results may change the interpretation of studies showing antiepileptic activity of CBX. The authors of this study concluded that suppression of epileptiform activity by CBX should not be regarded as uniquely caused by inhibition of gap junctions [35].

Thus, the experimental data on the antiepileptic effect of CBX are ambivalent. The antiepileptic effect of CBX can apparently be considered proven, but the manifestations of this effect can depend on a number of factors, and in some cases, to a large extent, leveled out. It is logical to assume that this effect may have a multifactorial biological basis. The aim of further research should be to elucidate all the elements of CBX action on the glial-neuronal complex.

CBX has been shown to reduce the epileptiform activity induced by 4-AP or cesium application or by exclusion of Mg^2+^ from the medium of hippocampal slices [36,37,38]. Systemic administration of CBX in vivo reduced the duration of pentylenetetrazole (PTZ)-induced chronic epileptic seizures [39]. The same effects were observed in audiogenic seizures in a genetic model of epilepsy in rats [10]. However, in all likelihood, systemic administration of CBX does not affect the number and duration of spike-wave discharges in a genetic animal model in the absence of epilepsy. These results, combined with ours, leave no doubt that CBX has significant EPI effects. It can be considered proven that this effect is achieved through its influence on GJs, but understanding the mechanism of this influence requires further research.

## 4. Materials and Methods

In total, 24 adult Wistar rats (male, 250–350 g, 3–5 months for in vivo, and 20–30 days for in vitro experiments) were used. Ten male rats, which were originally obtained from the Animal Resource Center, Universidad Central del Caribe (Bayamon, PR, USA) and maintained in the Animal Facility of the same institution, were used for in vitro experiments. Fourteen animals were used for in vivo experiments and were obtained from the Rappolovo Animal Facility, Russian Academy of Medical Sciences (St. Petersburg, Russia) and maintained in the Saint Petersburg State University animal facility. All procedures involving rodents were conducted in accordance with the National Institutes of Health (NIH) regulations concerning the use and care of experimental animals and approved by the UCC Institutional Animal Care and Use Committee (IACUC, for in vitro experiments, approval #10-XI-00) and the Ethical Committee for Animal Research of Saint Petersburg State University (for in vivo experiments, approval #131-03-4). All surgical procedures were performed using sterile/aseptic techniques in accordance with institutional and NIH guidelines, and the animals were anesthetized in all procedures involving surgery and before euthanasia.

### 4.1. In Vitro Experiments on Brain Slices

#### 4.1.1. Brain Slice Preparation and Patch-Clamp

In total, 10 rats at 30–60 days of age were rapidly decapitated. Hippocampal slices (400 μM) were prepared using a vibratome (VT1000S, Leica Microsystems GmbH, Wetzlar, Germany) in artificial cerebrospinal fluid (ACSF), containing (in mM) 127 NaCl, 2.5 KCl, 1.25 NaH2PO4, 25 NaHCO3, 2 CaCl2, 1 MgCl2, and 25 d-glucose, ice cold, saturated with a 95% O2/5% CO2 gas mixture at pH 7.4. A total of 16 slices from 10 different animals were used. Slices were perfused (0.1 mL/s) with the same ACSF at room temperature. For whole-cell recordings, membrane currents and voltages were measured with the single-electrode patch-clamp technique. Cells were visualized using an Olympus infrared microscope fixed on an X-Y stage (Narishige Int. Group, Tokyo, Japan) and equipped with differential interference contrast (model BX51WI, Olympus, Japan). Two piezoelectric micromanipulators (MX7500 with MC-1000 drive, Siskiyou, Inc., Grants Pass, OR, USA) were used for voltage-clamp and current-clamp recording. All manipulators and the microscope were separately fixed to an anti-vibration table (VH-AM, Newport Corporation, Irvine, CA, USA). A MultiClamp 700 A patch-clamp amplifier with a DigiData 1322 A interface (Molecular Devices, Inc., Sunnyvale, CA, USA) was used for recording and stimulation. The pClamp-10 software package (Molecular Devices, Inc., San Jose, CA, USA) was used for data acquisition and analysis. Borosilicate glass pipettes (O.D., 1.5 mm; I.D., 1.0 mm; World Precision Instruments, Sarasota, FL, USA) were pulled to a final resistance of 8–10 MΩ for astrocyte recordings in four steps using a P-97 puller (Sutter Instrument Co., Novato, CA, USA). Electrodes were filled with the following solution (in mM): 130 K-gluconate, 10 Na-gluconate, 4 NaCl, 4 phosphocreatine, 0.3 GTP-Na2, 4 Mg-ATP, and 10 HEPES, and the pH was adjusted to 7.2 with KOH. Astrocyte recordings were acquired only if the membrane potential (MP) was negative, up to −80 mV, and there was low input resistance (<20 MΩ) [14]. Experiments with a brain section and electrodes were performed using a difference in interference contrast (DIC) infrared video monitoring system (Figure 1C,D). Constant video monitoring, as well as constant control of electrophysiological parameters, allowed us to monitor cell patch conditions. A special perfusion system allowed us to rapidly apply different ACSF-based solutions containing 4-AP, CBX, or a mixture of the two. 

To record GJ currents between astrocytes, we patch-clamped two nearby astrocytes simultaneously in voltage-clamp mode and applied a meander voltage (+/−100 mV) to one of them while recording inverted currents in the other. This inverted current was recognized as the GJ current, summed from all GJs between the cells connected in the syncytium.

An additional isolated voltage amplifier (DP-301, Warner instruments, Holliston, MA, USA) and an additional manual MN4 manipulator (Narishige Int. Group, Tokyo, Japan) were used to connect a standard low-resistance electrode to record the local field potentials (LFPs) extracellularly (filtered at 0.1 Hz with a high-pass filter) in the slice.

#### 4.1.2. Chemicals and Materials

All chemicals and materials not specially mentioned were purchased from Sigma-Aldrich (Sigma-Aldrich, Saint Louis, MO, USA).

#### 4.1.3. Statistics and Measurements

GraphPad Prism 7.03 (GraphPad Software, Inc., La Jolla, CA, USA) was used for calculations of the Kolmogorov–Smirnov normality test, the ordinary *t*-test, and one-way analysis of variance (ANOVA) to determine statistical differences, as indicated for each experiment. Values were determined to be significantly different if the *p*-value was <0.05.

### 4.2. In Vivo Experiments

**Figure 5:**
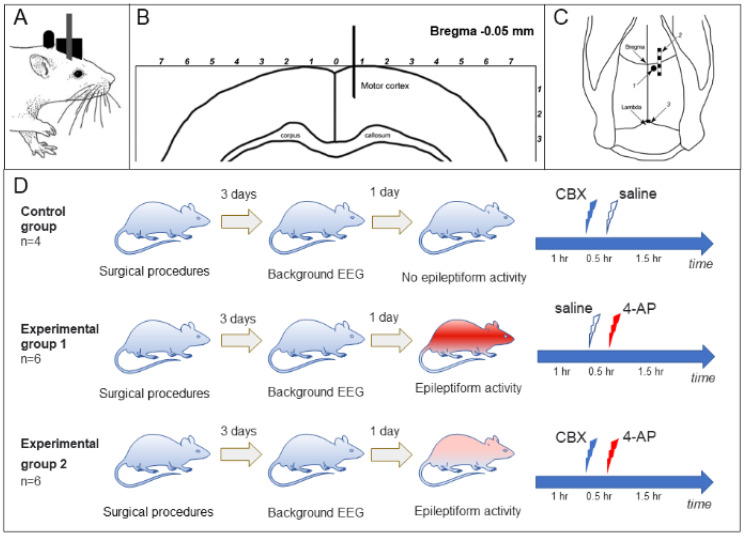
Experiment design: (**A**). The rat after surgical implantation; (**B**). The frontal view of the rat brain (modified from [41]); (**C**). The cannulas (1) and electrodes (2 and 3) location on the rat’s skull. (**D**). After surgical implantation of the electrodes and the cannula unit, the background ECoG was recorded, and analyzed 3 days later. CDX, the GJ blocker carbenoxolone, 50 mM, 4 µL; 4-AP, 4-aminopyridine, a K- channel inhibitor, 25 mM, 2 µL (for other explanations, see text).

For in vivo experiments,14 adult rats (Wistar male, 250–350 g, 3–5 months) were implanted with a block of electrodes for recording an electrocorticogram (ECoG) and a cannula for the intracortical introduction of substances [40]. The type of metal electrode block, as well as the electrode and cannula implantation procedure, was described previously [36]. Two blocks of ECoG intracranial electrodes were implanted under general anesthesia (100 mg/kg Zoletil intraperitoneally in combination with 0.2 mg/kg xylazine, intramuscularly); coordinates are given in millimeters relative to the bregma: AP = from +2.5 to –2.5 mm; L = ±1.5 mm, and the reference electrode (silver plate with an area of 2 mm^2^) was placed under the occipital bone at *p* = –8.5. Rats also were implanted with a stainless-steel guide cannula (g23, d = 0.6 mm), which was used to insert an injection cannula (g30, d = 0.3 mm). The cannula was inserted into the brain (AP = –0.5; L = 0.7) to a depth of 1.5 mm (Figure 5A–C).

The carbenoxolone (CBX) molecule is polar and relatively large, so it has very poorly crossing the blood–brain barrier [42]. Other studies show that CBX does not cross the blood–brain barrier at all [43]. This applies to both intraperitoneal and intravenous CBX. In addition, CBX is metabolized differently in different parts of the body, and together produces a large number of different metabolites [42]. In any case, the system administration of CBX is inadequate for studying the effects on the targets in the central nervous system. 4-Aminopyridine (4-AP) is used intravenously and orally to relieve symptoms of Eaton–Lambert syndrome, multiple sclerosis, Huntington’s chorea, Alzheimer’s disease, and some other neurological disorders [44]. However, to obtain focal epileptic seizures, an adequate dose of 4-AP must be injected locally into the brain tissue [45,46]. Thus, within the framework of our research task, there is no alternative to the methods of administration of CBX and 4-AP.

After surgical procedures, the rats were allowed to recover, and ECoG recording experiments were started no earlier than 3 days later. During ECoG recording, the rat was in the experimental box engaging in free behavior. Simultaneously with ECoG registration, video recording of the animal’s behavior was carried out. Episodes of sleep, wakefulness, grooming, exploratory activity, and convulsive states were noted.

The animals were divided into control (*n* = 4) and two experimental groups (Figure 5D): group 1 (“saline + 4-AP”, *n* = 6) and group 2 (“CBX + 4-AP”, *n* = 6). The rats of the control group received intracortical administration of saline (0.9% NaCl) or CBX (50 mM, 4 µL, Sigma-Aldrich, Saint Louis, MO, USA, diluted in 0.9% NaCl solution). Rats of the “saline + 4-AP” group (group 1) received intracortical administration of saline 30 min before the induction of epileptic activity by administration of 4-AP (25 mM, 2 µL, Sigma-Aldrich, USA). The animals of the “CBX + 4-AP” group (group 2) received intracortical administration of CBX (50 mM, 4 µL) 30 min before epileptogenic injection of 4-AP. 

Similar doses of 4-AP have been used by various researchers for intracortical administration in models of induced focal epilepsy, in vivo, and on neocortical slices [40,47,48]. CBX is administered at doses up to 100 mg/kg in various epilepsy models [49]. As shown by our and other works [11,40,50], the latent period of seizure development with cortical administration of 4-AP is 20 to 30 min. In the present work, we were interested in whether CBX administration would prevent the development of seizures or significantly attenuate them. Therefore, we chose a 30 min interval between CBX and 4-AP administration.

In animals of all groups, 3 days after implantation of electrodes, a 3 h background ECoG was recorded, which made it possible to exclude manifestations of spontaneous epileptiform activity and/or manifestations of post-traumatic epilepsy. One day after the background activity was recorded, an experiment with intracortical administration was carried out. In each experiment, at the beginning a background ECoG was recorded for 60 min. After that, intracortical administration of saline (group 1) or CBX (group 2) was carried out; 30 min later, 2 μL of 25 mM 4-AP was injected in both experimental groups, and the ECoG recording continued for another 1.5 h. 

The experimental setup consisted of an amplifier (x1000 gain, USF-8; Beta Telecom, St. Petersburg, Russia) and an L-791 (L-CARD) analog-to-digital converter. Data processing was carried out using Bioactivity Recorder v5.9.2 (Sibarov Biotechnologies, St. Petersburg, Russia) and Clampfit 10.2 (Molecular Devices, Toronto, Canada). Latent periods of onset and duration of episodes of spike-wave activity after 4-AP were analyzed in the two groups. Comparative statistical analysis of data was carried out using GraphPad Prism 7.03 software (GraphPad Software, Inc., La Jolla, CA, USA). We used the Kolmogorov–Smirnov test for data distribution normality and the two-tailed unpaired Mann–Whitney test for two-group comparison. Differences between groups were tested using two-way ANOVA. Values were determined to be significantly different if the *p*-value was <0.05.

## 5. Conclusions

As a result of the experiments carried out here, we can confidently say that premedication with CBX in the 4-AP model of epilepsy leads to a decrease in latency period and total seizure duration and also leads to prevention of the development of status epilepticus. Intracortical administration of CBX does not affect the instantaneous repetition rate of spike waves in seizures of epileptiform activity. Our in vitro results confirm that CBX reversibly blocks epileptiform activity in living rat hippocampal slices. Despite the fact that the full mechanism of suppression of epileptic activity by CBX remains unclear, it is highly likely that the astrocytic syncytium plays a role in this mechanism, and suppression of synchronized activity is mediated through an ultrahigh concentration of extracellular potassium. It is likely that specific regulators of GJ activity in astrocytes, especially those more selective then CBX, have great potential in antiepileptic therapy and should be the subject of future studies.

The content is solely the responsibility of the authors and does not necessarily represent the official views of the founders.
